# Pathogenesis and Clinical Relevance of *Candida* Biofilms in Vulvovaginal Candidiasis

**DOI:** 10.3389/fmicb.2020.544480

**Published:** 2020-11-11

**Authors:** Carmen Rodríguez-Cerdeira, Erick Martínez-Herrera, Miguel Carnero-Gregorio, Adriana López-Barcenas, Gabriella Fabbrocini, Monika Fida, May El-Samahy, José Luís González-Cespón

**Affiliations:** ^1^Efficiency, Quality, and Costs in Health Services Research Group (EFISALUD), Health Research Institute, SERGAS-UVIGO, Vigo, Spain; ^2^Department of Dermatology, Hospital do Meixoeiro and University of Vigo, Vigo, Spain; ^3^European Women’s Dermatologic and Venereologic Society, Tui, Spain; ^4^Psychodermatology Task Force of the Ibero-Latin American College of Dermatology (CILAD), Buenos Aires, Argentina; ^5^Unidad de Investigación, Hospital Regional de Alta Especialidad de Ixtapaluca, Ixtapaluca, Mexico; ^6^Department of Molecular Diagnosis (Array & NGS Division), Institute of Cellular and Molecular Studies, Lugo, Spain; ^7^Section of Mycology, Department of Dermatology, Manuel Gea González hospital, Mexico City, Mexico; ^8^Department of Dermatology, University of Naples Federico II, Naples, Italy; ^9^Department of Dermatology, University of Medicine, Tirana, Tirana, Albania; ^10^Department of Dermatology, Faculty of Medicine, Ain Shams University, Cairo, Egypt

**Keywords:** vulvovaginal candidiasis, *Candida* spp., biofilm models, proteomic, genomic, new anti-*Candida* targets

## Abstract

The ability of *Candida* spp. to form biofilms is crucial for its pathogenicity, and thus, it should be considered an important virulence factor in vulvovaginal candidiasis (VVC) and recurrent VVC (RVVC). Its ability to generate biofilms is multifactorial and is generally believed to depend on the site of infection, species and strain involved, and the microenvironment in which the infection develops. Therefore, both cell surface proteins, such as Hwp1, Als1, and Als2, and the cell wall-related protein, Sun41, play a critical role in the adhesion and virulence of the biofilm. Immunological and pharmacological approaches have identified the NLRP3 inflammasome as a crucial molecular factor contributing to host immunopathology. In this context, we have earlier shown that *Candida albicans* associated with hyphae-secreted aspartyl proteinases (specifically SAP4-6) contribute to the immunopathology of the disease. Transcriptome profiling has revealed that non-coding transcripts regulate protein synthesis post-transcriptionally, which is important for the growth of *Candida* spp. Other studies have employed RNA sequencing to identify differences in the 1,245 *Candida* genes involved in surface and invasive cellular metabolism regulation. *In vitro* systems allow the simultaneous processing of a large number of samples, making them an ideal screening technique for estimating various physicochemical parameters, testing the activity of antimicrobial agents, and analyzing genes involved in biofilm formation and regulation (*in situ*) in specific strains. Murine VVC models are used to study *C. albicans* infection, especially in trials of novel treatments and to understand the cause(s) for resistance to conventional therapeutics. This review on the clinical relevance of *Candida* biofilms in VVC focuses on important advances in its genomics, transcriptomics, and proteomics. Moreover, recent experiments on the influence of biofilm formation on VVC or RVVC pathogenesis in laboratory animals have been discussed. A clear elucidation of one of the pathogenesis mechanisms employed by *Candida* biofilms in vulvovaginal candidiasis and its applications in clinical practice represents the most significant contribution of this manuscript.

## Introduction

Vulvovaginal candidiasis (VVC) is a usual fungal infection caused by *Candida* species, mainly *Candida albicans*. It is characterized by inflammatory signs and symptoms detected in the vulva and vaginal mucosa that are caused and linked by an overgrowth of *Candida* species, which are generally present as quiescent vaginal commensals (Sobel, 2007).

The genus *Candida* belongs to the *Saccharomycetaceae* family. The organisms reproduce asexually or anamorphically through blastoconidia, do not produce melanin pigments, have diverse morphologies (globose, oval, cylindrical, and elliptical), and are sized between 3 and 10 μm. In humans, different species of this genus are identified as commensals of the gastrointestinal tract, upper respiratory tract, skin, oral, vulvar, and vaginal mucosa (Neppelenbroek et al., 2014).

Comparative *Candida* species genomics will enhance our understanding of the genetic and phenotypic variations that occur inside the vulva and vagina and will further facilitate better understanding of the pathogenesis of these commensals in VVC (Bradford et al., 2017).

Despite the fact that *C. albicans* is the most common VVC-causing pathogen, the identification of non-*C. albicans Candida* (NCAC) species, mainly *C. glabrata*, as the originator of this infection appears to be continuously increasing. It is difficult to determine its prevalence because the diagnosis and treatment are often based on the symptoms and not dependent on the confirmation by microscopic examination or using culture techniques (Mahmoudi Rad et al., 2011).

**FIGURE 1:**
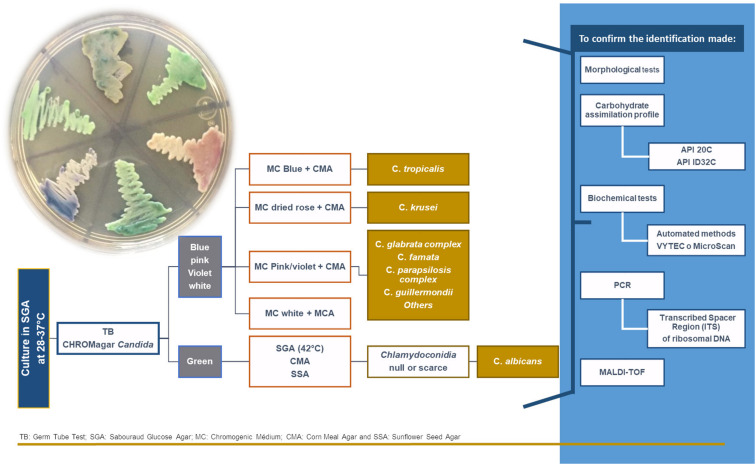
The algorithm used for the identification of *Candida* yeasts.

Other NCAC species that must be taken into account are *C. tropicalis*, *C. parapsilosis*, *C. kefyr*, *C. krusei*, *C. guilliermondii*, *C. famata*, and *C. lusitaniae*. The production of virulence factors by these strains depends on the site and degree of invasion, as well as the nature of host response. Early identification of the involved strain is, therefore, essential for rapid diagnosis (Bitew and Abebaw, 2018). The techniques used most frequently for their identification are shown in Figure 1.

Yeasts that are present in the vagina become pathogenic when the host vagina allows it (Miró et al., 2017). Host-related factors involve pregnancy (Brown et al., 2019), hormonal imbalance, ill-treated diabetes, immunosuppression (either through immunosuppressive drugs or infection with the human immunodeficiency virus), which can act as a predisposing factor (Yano et al., 2019), use of broad-spectrum antibiotics (Shukla and Sobel, 2019) and glucocorticoids, and genetic predispositions (Gonçalves et al., 2016).

Other less recognized or more debatable factors are oral contraceptive administration (especially when the estrogen dose is high), estrogen therapy, use of intrauterine devices, spermicides, and condoms, and certain hygiene, clothing, and sexual practice-related routines (Corsello et al., 2003; Maraki et al., 2019). In general, the most common clinical characteristic related to vaginal inflammation is itching, which is followed by burning.

Recurrent vulvovaginal candidiasis (RVVC) is featured by four or more episodes of symptomatic infection in the same year, and clearly affects the quality of life in women (Blostein et al., 2017). Djohan et al. (2019) have reported a prevalence of RVVC in 23.5% (CI95: 19.49–28.02%) of women of reproductive age and identified five *Candida* species in a group of 94 patients with RVVC: *C. albicans* (59.6%), *C. glabrata* (19.1%), *C. tropicalis* (16%), *C. krusei* (4.2%), and *C. inconspicua* (1.1%). However, other authors have provided lower figures, but there is a consensus that the frequency of RVVC is increasing (Gonçalves et al., 2016; Denning et al., 2018).

Among other species, *C. kefyr* is frequently found to be present in the polyfungal population (Ozcan et al., 2010; Guzel et al., 2011).

However, a new pathogen, *C. auris*, has emerged recently (Kumar et al., 2015). Another emerging pathogen that has been identified is *C. nivariensis*, which is genetically related to *C. glabrata*. *C. nivariensis* was reported for the first time in Canary Islands, Spain, and at present, it seems to widely distributed worldwide. Inhibition of quorum sensing molecules with natamycin against *C. tropicalis* haven been used successfully by researchers (Agustín et al., 2019). However, the number of cases is low possibly due to the impossible phenotypic identification of *C. glabrata* (Aznar-Marin et al., 2016).

New compounds have been developed, with more candidates in the pipeline, for the treatment of biofilms associated with *Candida* spp. infections. Some of these are naturally occurring, while others have been developed by synthetic routes optimized in the laboratory. New antifungals such as acridone (de Oliveira et al., 2019) or nanotechnology-based techniques, such as the encapsulation of citric acid into Mg-Al-layered double hydroxides, have shown optimistic trends.

Prebiotics, probiotics, and symbiotics also offer promising results, as these are related to the stimulation of host immunity and their presence is known to decrease the prevalence of *Candida* spp. (Davani-Davari et al., 2019).

Plant extracts and essential oils derived from the leaves of plants of Brazilian origin described by Costa et al. (2017), such as *Hymenaea courbaril* var. *courbaril*, *Myroxylon peruiferum*, and *Vismia guianensis*, have shown therapeutic potential in this regard. The derivatives of flavonoids, such as methylated isoflavones (i.e., formononetin 7-*O*-apiosyl glucoside) (Martins et al., 2016), or the polyphenol, licochalcone-A, found in the roots of *Glycyrrhiza* spp. (Seleem et al., 2016) or honey (Fernandes et al., 2020), have also been demonstrated to be useful.

Photodynamic therapy (Ghasemi et al., 2019), quorum sensing molecules and antibodies/peptides (Carrano et al., 2019) have emerged as viable treatment modalities in recent years. More sophisticated techniques, such as lock therapy (Visek et al., 2019), represent an important potential breakthrough in the treatment of biofilms produced due to infection by *Candida* spp.

In this review, we aimed to discuss the main characteristics of the female vulvovaginal mucosa, and the mechanisms employed by *Candida* spp. to colonize the host. Furthermore, the different kinds of biofilms formed by *Candida* spp., their impact on clinical practices, and the development of new agents against them will also be reviewed in depth.

## Materials and Methods

**FIGURE 2:**
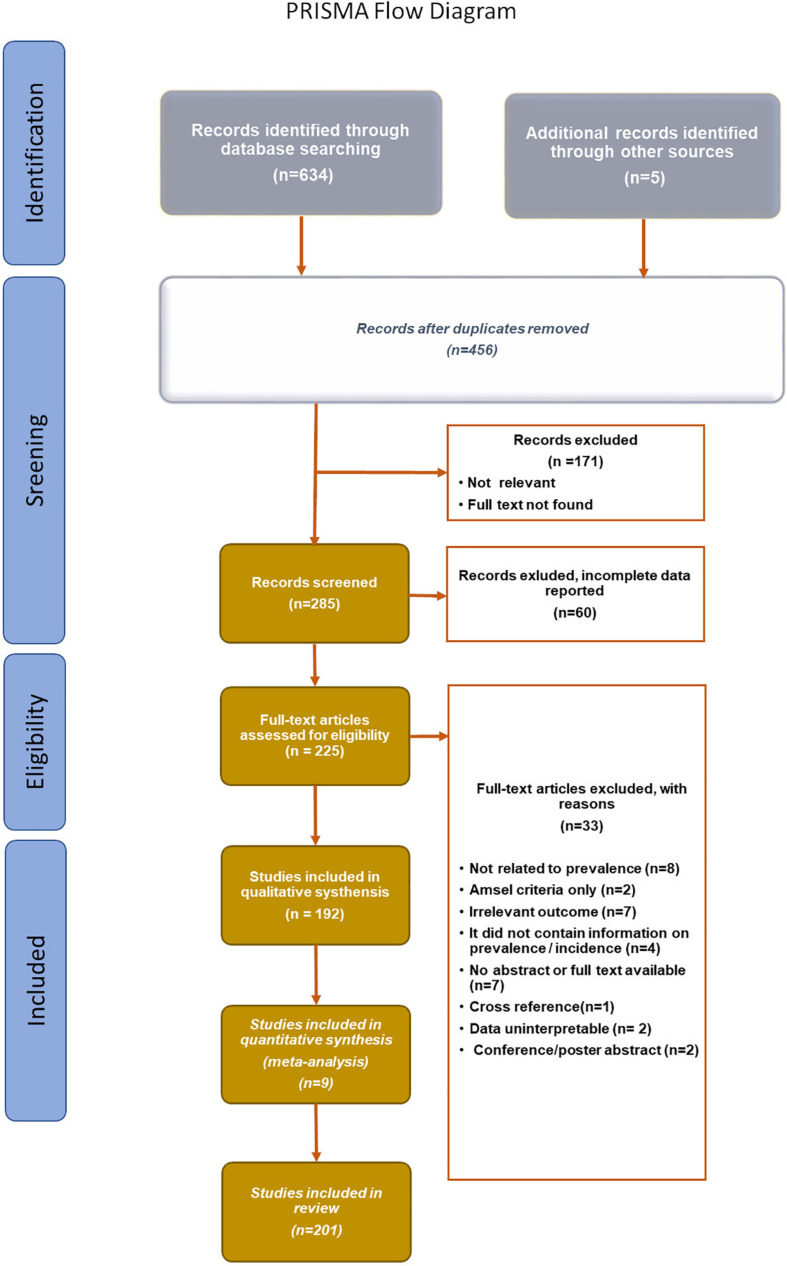
PRISMA Flow diagram (Moher et al., 2015).

The databases MEDLINE (PubMed), and Embase were searched extensively for articles published from January 2003 to January 2020, using the following search terms: vulvovaginal candidiasis, *Candida* spp*., C. albicans, C. glabrata, C. parapsilosis, C. tropicalis, C. kefyr, C. auris*, *C. nivariensis*, biofilm, and antifungal agents. PRISMA Flow Diagram (Figure 2) depicted the flow of reports through the different stages of this systematic review. It mapped the number of records that were identified, included or excluded, and the reasons for exclusions.

## Characteristics of the Vulvovaginal Mucosa and Mechanisms That *Candida* spp. Deploys Colonize It

### Vaginal Ecosystem

The vaginal microbiota, which mainly constitutes of *Lactobacillus* spp., forms the most important defensive barrier against candidal infections (Smith and Ravel, 2017). The vaginal ecosystem balance is maintained at the expense of articulated interactions of different mechanisms, which keeps the female genital tract (FGT) healthy. Both epithelial cells of the vaginal mucosa and FGT immune system are under the control of the female sex hormones, estradiol, and progesterone. On the vaginal epithelium, estrogens regulate trophism, vascularity, and tissue vitality; in addition, it influences conditions such as humidity, pH, and vaginal discharge as well as vaginal microbiota composition (Achilles et al., 2018).

*Candida* spp. is considered as opportunistic pathogens. Although initially it was thought that yeast participates passively in the pathogenesis process and fungal infection establishment, but recently this concept has been modified, proposing an active participation of these microorganisms via the action of virulence factors (El-Houssaini et al., 2019).

Factors contributing to *C. albicans* pathogenesis include morphogenesis (transition between single-celled yeast cells to filamentous growth forms), secretion of enzymes like aspartyl proteases (SAP) and phospholipases, and host recognition biomolecules (adhesins), which lead to the biofilm formation process. Likewise, the phenotypic change is accompanied by alterations in antigen expression, colonial morphology, and affinity of *C. albicans* to tissues (El-Houssaini et al., 2019; Monika, 2019).

The experimental information on the contribution of these virulence factors has revealed that their individual participation is not sufficient to explain the mechanisms of damage in the host; moreover, there is a combined regulation mechanism operating between them. This was further demonstrated with polymorphism and gene expression analyses of SAPs. The studies currently being carried out on the genetic expression of virulence factors, which depends on different environmental conditions, will allow us to better understand how the biological activity of *Candida* spp. modifies to favor adhesion or penetration processes and, consequently, modifies its role to become a pathogenic microorganism (Silva et al., 2012; Kalaiarasan et al., 2018; Willems et al., 2018).

Araújo Paulo, de Medeiros et al. (2017) reported that the virulence characteristics of *C. albicans* contributing to its pathogenicity include the skill to adhere to human epithelial and endothelial cells, yeast to hyphae transition, extracellular hydrolytic enzyme (proteinases and phospholipases) secretion, phenotypic adaptability, and biofilm formation. Further, there is a relationship between virulence factor expression and VVC signs and symptoms identified in the patients, but it does not seem to be essential for the transition from colonization to infection.

### Adherence

Adhesion is initiated by non-specific binding, which is based on attractive and repulsive forces that bring the pathogen close to the host surface. Adhesins are biomolecules that promote the binding of specific ligands to host cells. The investigations by Zhao et al. (2003) stake the adhesion about the family of adhesins called ALS (Agglutinin-Like Sequence), which belongs to a group of eight genetically related glycosylated proteins with great allelic variability (ALS1–ALS7 and ALS9). ALS1 and ALS3 are particularly found to be important in the adhesion process. Other molecules that also promote the adhesion and penetration of *C. albicans* into the tissues are the polysaccharides, proteins, and lipids present on cell surface. Data supplied by the *C. albicans* genome sequencing project provided the main indication that the strain SC5314 encodes two different ALS9-like sequences and three ALS genes (ALS5, ALS1, and ALS9) next to chromosome 6 (Araújo et al., 2017).

Wilkins et al. (2018) showed that the eukaryotic proteome contains certain components that are encoded by open reading frame (ORF)s possessing protein-coding tandem repeat (TRs) (TR-ORFs, pcTRs), but their biological consequences are not clearly known. Ichikawa et al. (2019) analyzed the adherence and cytotoxicity of *C. glabrata* that selectively adhered to the epithelial cells. On the contrary, *C. parapsilosis* showed poor adherence to the HaCaT keratinocytes. *C. glabrata* caused more damage to the A549 cells than to the HaCaT cells, suggesting that *Candida* spp. exhibits different effects depending on the tissue on which they can adhere.

Modrzewska and Kurnatowski (2015) demonstrated that the adherence of *Candida* spp. on the tissue or cell wall of a host is based on the relationship that might exist between the two. A variety of genes that are responsible for the adhesion capacity of fungal cell wall, like HWP1, which encodes protein 1 of the hyphal wall and germ tubes and is responsible for biofilm formation. It is also responsible for the fungal virulence capacity and therefore, provides resistance to antifungal agents. This gene is identified in both oral cavity and vaginal yeast infections (Fan et al., 2013).

Ardehali et al. (2019) identified that the frequency of HWP1 gene among *C. albicans* was 95%, with HWP1 being the most detected virulence factor, and SAP4 being the least detected one in the clinical specimens collected from patients hospitalized in the Intensive Care Unit (ICU) of Milad hospital, Tehran, Iran.

According to Cota and Hoyer (2015), the ALS genes encode fungal glycoproteins on the cell surface. A total of eight ALS genes have been reported so far (ASL1–7 and ALS9). According to Liu and Filler (2011), the ALS3 gene exhibits a dual function of adhesin and invasion. ALS3 is used for the preparation of vaccines because of its level of *in vivo* dispersion. According to Zajac et al. (2016), other genes that have been involved in cell adhesion are EPA (epithelial adhesin) genes that are specifically identified in *C. glabrata*, as in this *Candida* sp., ALS genes have not been identified to date.

According to Modrzewska and Kurnatowski (2015), the most important adhesins present on the fungal cell wall in *Candida* spp. are: ALS, EPA, HWP1, but also EAP1, SUN41, CSH1 and probably HYR1; for significant adhesion, they also possess Sap (secreted aspartyl proteases). Various other genes reported to positively affect adhesion and hyphal formation are CZF1, EFG1, TUP1, TPK1, TPK2, HGC1, RAS1, RIM101, VPS11, ECM1, CKA2, BCR1, BUD2, RSR1, IRS4, CHS2, SCS7, UBI4, UME6, TEC1, and GAT2.

### Morphogenesis

Morphogenesis refers to the conversion of a particular yeast form (unicellular) to the filamentous form of the fungus (hypha or pseudohypha), making it possible to adapt to different biological niches, which favors the fungal spread.

According to Min et al. (2019), *N*-acetylglucosamine (GlcNAc) potently induces the transition of *C. albicans* from budding to filamentous hypha growth. It also stimulates an epigenetic change that converts the white cells to opaque cells, which vary in morphology, metabolism, and virulence properties.

According to Min et al. (2018), the presence of TF (transcription factor) Ndt80 is necessary for the growth of hyphae in *C. albicans* and its presence is significant since it is conserved in most of the fungal species; however, it must be taken into account that its quantity is variable among the different fungal species. It regulates a variety of processes, such as sexual development, resistance to antifungals, fibrillation, virulence, and nutritional stress response, among others.

The human fungal pathogen *C. albicans* contains three virulence genes, namely NDT80, REP1, and RON1. RON1 deletion leads to growth defects when grown in GlcNAc media and hypha induction. To avoid this, a new short cross-linked palindromic/CRISPR repeat associated with Cas9 has been used for gene deletions (Min et al., 2019).

### Phenotypic Switch

Tang et al. (2019) demonstrated that the majority of *C. albicans* strains are capable of undergoing the phenomenon of phenotypic switch, which is associated with the manifestation of phenotypic changes at high frequency and a consequence of the action of numerous environmental factors. This phenomenon induces an epigenetic change in colonial morphology. Hu et al. (2016) collected and subsequently employed 231 clinical isolates for genotyping as well as phenotypic switch analysis. A total of 65 different genotypes were recognized by microsatellite locus (CAI) genotyping assay, and some prominent genotypes were identified from certain human niches. The authors established that there is an association between the phenotypic switching and genotypes of the CAI microsatellite locus in these *C. albicans* clinical isolates.

During this process, enzymes able to break down the structural polymers that provide accessible nutrients for fungal growth, capability to evade the immune system, and suppress the host pro-inflammatory response are released. They are also responsible for inactivating certain molecules that are directly linked to the host defense mechanisms (Hu et al., 2016). The main extracellular enzymes (Basmaciyan et al., 2019) that are related to the pathogenesis of *Candida* spp. are described below:

Secreted aspartic proteinases (Sap) (Kadry et al., 2018) are regarded as one of the most critical virulence factors as they are related to adhesion, invasion, tissue damage, and evasion of the host immune system, due to its ability to hydrolyze various host proteins, including albumin, keratin, collagen, fibronectin, interleukin 1β (IL-1β), and Immunoglobulin A.

### Phospholipases

Studies by Richmond and Smith (2011) and Dabiri et al. (2018) demonstrated the existence of glycoproteins with hydrolase activity (glyphospholipid esters are hydrolyzed) and lysophospholipase transacylase (release fatty acids from lysophospholipids and transfer free fatty acid to another phospholipid), which play key roles in the infection process.

## Biofilms

For a long time, investigations in the area of microbial biofilms have received increasing momentum and scientific evidence has led to a change in the way we consider microbial life (de Barros et al., 2020). Biofilms are defined as heterogeneous and dynamic microbial communities undergoing continuous transformation. They can be comprised by a single bacterial/fungal species, or can be polymicrobial. Since there are differences between biofilms formed on mucosal and abiotic cells, it is precise to have *in vitro* and *in vivo* models that can mimic these processes (Tournu and Van Dijck, 2012).

In this context, new tools to analyze *Candida* biofilms have been implemented, including genomic, transcriptomic, proteomic, and metabolomics approaches, using a wide variety of animal models.

According to Cavalheiro and Teixeira (2018), biofilm formation is a complicated process, which starts with adhesion on an abiotic surface, a tissue, or the air–liquid interface. It is a continuous process, which undergoes different stages of development: (a) conditioning, (b) adhesion, (c) quorum sensing-induced extracellular matrix synthesis, (d) maturation, and (e) dispersion.

These phases lead to the formation of a uniform structure in the form of homogeneous deposits and cellular viscous accumulations surrounded by a matrix of polymers with open channels for water movement (Chandra and Mukherjee, 2015).

Cells in the biofilms have different properties than those of isolated cells, which is because they have low growth rates and great resistance to antimicrobial treatment, and it makes their behavior different from those of planktonic cells (Muzny and Schwebke, 2015). Biofilms are biological systems that interact and evolve together. Hence, they need a wide network of genetic regulation to carry out both intra- and inter-cellular communication and specialization and originate biofilms made up of the same species or multi-species (Rodríguez-Cerdeira et al., 2019). *C. albicans* biofilm formation is a complex process that begins when the yeast cells adhere to host tissue surface, and the biofilm starts to form at an early stage (8–11 h), undergoes an intermediate stage (12–30 h), and finally reaches the mature stage (38–72 h) (Mathé and Van Dijck, 2013).

**FIGURE 3:**
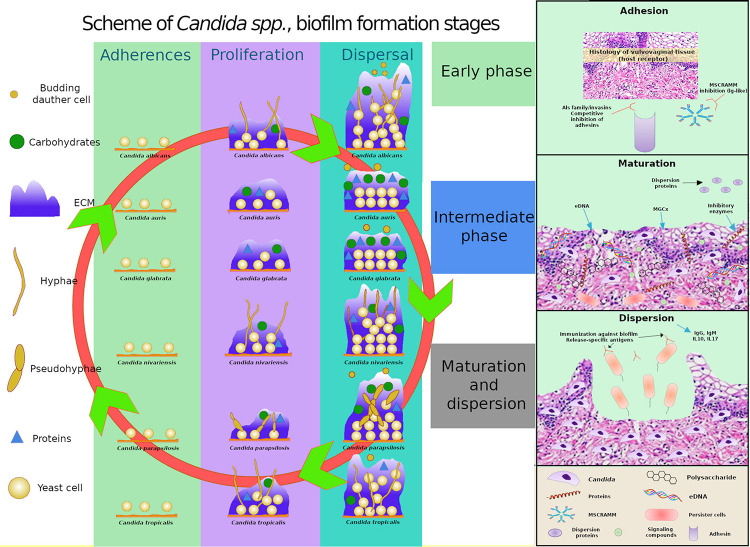
Comparative schematics of three stages of biofilm formation in *C. albicans, C. glabrata, C. tropicalis, and C. parapsilosis, C. auris and C. nivariensis* highlighting the different capacities of the species in producing extracellular matrix (ECM), the varying components present in the ECM, and the ability of the species to exhibit different cell morphologies, and highlights of the transcriptional factors involved in adhesion, extracellular polymeric substances, filamentation, and biofilm formation. MSCRAMM, microbial surface components recognizing adhesive matrix molecules; MGCx, extracellular matrix mannan-glucan complex; eDNA, external DNA.

The mature biofilm comprises a dense network of yeasts, hyphae, and pseudohyphae, which is covered by an extracellular matrix and frequently associated with bacteria, as also described by Silva et al. (2012, 2014). Each *Candida* species (*C. albicans*, *C. glabrata*, *C. parapsilosis*, *C. tropicalis*, *C. nivariensis*, *C. kefyr*, and *C. auris*) shows significant peculiarities in terms of biofilm formation, which results in different morphologies, extracellular matrix composition, and resilience to antifungal agents (Cavalheiro and Teixeira, 2018) (Figure 3).

The importance of certain virulence factors was found to be closely related to Sap, which is related to biofilm production as described by Kadry et al. (2018). Isolates that were found to be resistant to three different anti-fungal drugs were reported as strong biofilm producers and shown to possess proteolytic activity as they contained either Sap9 or Sap10, or both (Černáková et al., 2019).

El-Houssaini et al. (2019) identified the virulence factors related to hydrolases, which are involved in cell surface hydrophobicity and biofilm production in vaginal samples. *C. albicans* resistance and virulence patterns observed in the samples were variable. The samples were highly resistant to physical and chemical treatments, making it difficult to treat them (Marak and Dhanashree, 2018).

### Genomics of the Biofilm

Numerous genes involved in biofilm formation have been identified in the *C. albicans* genome, including 50 transcriptional regulatory genes, and 101 transcriptional non-regulatory genes (Shukla and Sobel, 2019). Nobile et al. (2012) described the six main transcriptional regulatory genes known as “master” transcriptional regulators, including Efg1 (enhanced filamentous growth), Tec1 (transposon enhancement control), Bcr1 (biofilm and cell wall regulator), Ntd80 (meiosis-specific transcription factor), Brg1 (biofilm regulator), and Rob1 (biofilm regulator). Each of these genes are mandatory for normal biofilm development and have been identified both *in vitro*, under standard laboratory conditions and *in vivo*, in laboratory animals (rats).

Apart from these 6 genes, 44 other transcriptional regulatory genes have also been identified, and the deletion of these genes has been associated with erroneous biofilm formation (Ni et al., 2009). Chatterjee et al. (2015) identified eight OPT genes encoding the oligopeptide transporters that help *C. auris* to adapt to different host tissues. They also found orthologs for these genes that were believed to carry hexose, maltose, and permeases (amino acid permeases, sulfur permeases, allantoic permeases, glycerol permeases, and iron permeases), which also help the fungi to adapt and live in their host. Other transcriptional regulators have also been recently found to be involved in adherence (Finkel et al., 2012).

The first step in biofilm formation process is surface adhesion, in which the *Candida* cell wall interacts with the host cell surfaces. This process involves a series of genes that encode several proteins, such as ALS adhesins, of which ALS1, ALS3, and ALS5 are implicated in the adhesion process; Eap1, regulated by the factor EFG1; HWP1, which promotes the fixation of *Candida* cells to the host surface and is a potential pathogenicity factor (it is absent in *C. glabrata*); PGA10 and PBR1—it has been shown that when the PBR1 gene is deleted, the adhesion of *C. albicans* to blood cells decreases; EPA family of genes, of which EPA1, EPA6, and EPA7 are the main epithelial adhesins, and their deletion hinders or decreases adhesion to surfaces; AWP adhesins, whose expression is higher in biofilms than in plant cells (Orsi et al., 2014; Valotteau et al., 2019).

Inglis and Sherlock (2013) showed that the expression of these wall proteins is regulated by two main genes: BCR1 and TEC1. TEC1 regulates BCR1 via cell signaling cascades and is involved in yeast differentiation into hypha. EPAs (epithelial adhesin) are specific for *C. glabrata* and the genes encoding ALS proteins have not been observed in mucosa yet.

CKA2, BCR1, BUD2, RSR1, and HWP1 are the genes that modify adhesion and tissue damage, but not invasion, in the host. Other genes that positively affect adhesion and hyphal formation have been reported, including CHS2 (synthesizes chitin), SCS7 (involved in cell membrane sphingolipid formation), UBI4 (contributes to ubiquitination), and genes that participate in adhesion and filamentation. UME6, TEC1, and GAT2 are involved in subsequent biofilm formation (Modrzewska and Kurnatowski, 2015).

The importance of the adherence regulatory genes (Bcr1, Ace2, and Snf5) lies in the fact that they are necessary for biofilm formation *in vitro* (Guan et al., 2013; van Wijlick et al., 2016; Burgain et al.; 2019).

After the initial adhesion of round yeast cells to the host surface to form a basal layer, the next phase of biofilm establishment is the growth and proliferation of hyphal cells. During the *C. albicans* proliferation phase, it multiplies and forms a monolayer adhesion zone where filamentation occurs, with the hyphae leading to general stability and acting as a support for the different cell morphologies: yeast, pseudohypha, hypha, and other cells of microorganisms that might be a part of the biofilm (Chandra and Mukherjee, 2015). According to McCall et al. (2018) and Min et al. (2018), hyphal growth in *C. albicans* is dependent on the presence of TF (transcription factor) Ndt80, which is responsible for regulating a variety of processes, including sexual development, resistance to antifungals, fibrillation, virulence, and nutritional stress response, among others. Hence, *C. albicans* possesses three paralogs (genes of different species, which descends to a single gene by genetic duplication in evolution) of NDT80 (NDT80, RON1, and REP1), and each of them provides *C. albicans* with specific characteristics. NDT80 is involved in resistance under different stress conditions, as well as in hyphal growth, and RON1 deletion has *shown to cause growth defects when C. albicans* is grown in GlcNAc media.

Glazier et al. (2017) highlighted that biofilm maturation is regulated by several transcription factors in *C. albicans*, including BCR1, EFG1, TEC1, NDT80, ROB1, and BRG1. When any one of them is deleted, biofilms are not formed correctly. Other transcription factors, such as CZF1, GZF3, UME6, CPH2, and ACE2, are also shown to be involved in *C. parapsilosis* biofilm formation. Biofilm formation decreases dramatically if ACE2 is deleted, and it also regulates biofilm development in *C. albicans*; however, in *C. glabrata*, the ability to cause disease is increased when ACE2 is inactive.

Ng and Dean (2017) reported that the increased expression and maturation of sgRNA facilitates and improves the CRISPR/Cas9 mutagenesis in *C. albicans* by about 10 times. This may help us edit the *C. albicans* genome. Finally, REP1 has been shown to be negatively involved in the expression of the MDR1 drug flow pump, which provides resistance against several microorganisms (Chen et al., 2009).

In the maturation phase, biofilm development continues via morphological modifications, an increase in the number of cells, and extracellular polysaccharide matrix production. This extracellular matrix mediates adhesive and cohesive interactions, grants mechanical stability and integrity, allows cell dispersion, limits the diffusion of toxic substances and nutrients, and even acts as an enzyme system. However, the main function of the extracellular matrix is to protect the biofilm from the important clinical repercussions, acting as a physical barrier to protect the biofilm cells from environmental factors (Chandra and Mukherjee, 2015; Rodríguez-Cerdeira et al., 2019).

Apart from the composition of the matrix, several recent studies have focused on its genetic regulation. *C. albicans* biofilm matrix *in vivo* and *in vitro* models were biochemically analyzed by Flemming and Wingender (2010) to identify the macromolecular components. The composition was as follows: 55% proteins and their glycosylated counterparts, 25% carbohydrates, 15% lipids, and 5% non-coding DNA. α-1,2-branched α-1,6-mannans were the most frequent polysaccharides related to unbranched α-1,6-glucans, forming a mannan–glucan complex on the matrix. However, they were a smaller proportion of extracellular matrix (ECM) components in *C. albicans* than β-1,3-glucan and β-1,6-glucan (Zarnowski et al., 2014).

Mitchell et al. (2015) and Dominguez et al. (2018) highlighted the importance of mannan and β-1,6-glucan in ECM formation. The elimination of proteins involved in ECM formation by deleting any one of the seven genes that governs the level of matrix mannan and β-1,6-glucan (ALG11, MNN9, MNN11, VAN1, MNN4-4, PMR1, and VRG4 for mannan production; BIG1 and KRE5 for β-1,6-glucan production) resulted in complete disappearance of the biofilm. In *C. parapsilosis*, the main function of Efg1 is to behave as a regulatory switch and participate in biofilm formation. Its absence leads to the production of an incomplete biofilm (Connolly et al., 2013).

In *C. albicans*, Cph1 acts as the terminal transcription factor of the MAP kinase pathway (Freire et al., 2018). This protein belongs to the family of the STE type transcription factor, *which is also present in other Candida* spp. (Bandara et al., 2013).

Brg1 controls the growth of hyphae in *C albicans*. When it is overexpressed, there is an increase in the expression of hyphae-specific genes, along with ALS3, HWP1, and ECE1 (Su et al., 2018). In *C. parapsilosis*, a homolog of Brg1 has been identified (Holland et al., 2014). According to Mendelsohn et al. (2017), the transcription factor Ume6, together with Nrg1 and Rfg1 decreases the expression of hypha specific genes ALS3, ECE1, and HWP1 in both *C. albicans* and *C. tropicalis*.

The cell surface proteins encoded by HYR1, ECE1, RBT5, ECM331, HWP1, ALS3, ALS1, and ALS9 genes are all regulated by Bcr1 gene. Upon overexpression of ALS3 in a bcr1/bcr1 cell, the biofilm formation phenotype was shown to be completely restored (Freire et al., 2018).

Vandeputte et al. (2012) identified that Rca1/Cst6 in *C. albicans* regulates hypha formation by facilitating the expression of GWP1, ECE1, HGC1, and ALS3 genes and the transcription factor Efg1. Cst6 is also a transcriptional controller of biofilm growth. It is a bZIP transcription factor, which belongs to the ATF/CREB family that inhibits the expression of the EPA6 gene and encodes an adhesion protein in the *C. glabrata* biofilm (Pohlers et al., 2017).

In *C. glabrata*, Leiva-Peláez et al. (2018) demonstrated that the expression of several related genes is subjected to subtelomeric silencing. HYR1, EPA1, EPA2, EPA3, EPA4, EPA5, EPA6, *and EPA7 genes of C. glabrata* undergo subtelomeric silencing due to their loci proximity to a telomere. The Sir complex (Sir2–Sir4), Rap1, Rif1, yKu70, and yKu80, as well as the Swi/Snf complex are shown to be involved in regulating biofilm formation.

Deletion of the PMR1, KRE5, and FKS1 genes was associated with the increased susceptibility to fluconazole (Navarro-Arias et al., 2016; Tanaka et al., 2016; Jiang et al., 2018). Chowdhary et al. (2018) demonstrated that persistent cells cultured from the biofilm exhibit higher expression of CDR genes (Khosravi Rad et al., 2016). According to Feng et al. (2016), a set of isogenic *C. albicans* strains carrying single or double deletions in genes encoding efflux pumps (Δcdr1, Δcdr2, Δmdr1/drc2, and Δmdr1/cdr1) show antifungal resistance with paired strains. Tan et al. (2018) demonstrated that β-1,3-glucan has a crucial role in *Candida* biofilm formation and stress response in biofilm-forming *Candida*. They also showed that β-1,3-glucanase might be useful as an anti-biofilm agent.

In contrast, there are two major regulators of ECM production: Rml1 and Zap1. It has been shown that Rm11 deletion leads to the reduction in matrix levels (Nett et al., 2011; Pierce et al., 2017). However, Zap1 deletion leads to an increase in matrix levels, which is due to the overexpression of the glucoamylases, Gca1 and Gca2, as these enzymes, together with other hydrolyzing enzymes, are important for biopolymer degradation (Gulati and Nobile, 2016). Previous studies revealed that Δzap1 mutant, which is defective in the expression of zinc regulator Zap1, enhances the accumulation of yeast cells in biofilms (Ganguly et al., 2011).

In the late or dispersal phase, unbound *Candida* cells scatter from the biofilm and attempt to colonize other surfaces. Thus, it is possible for the cells to initiate the formation of new biofilms or spread in the host tissues, which ultimately leads to the development of candidemia and disseminated invasive diseases. Dispersed cells exhibit strong adherence, capacity to form new biofilms, and virulence. This process depends on three identified transcriptional regulators: Pes1, Nrg1, and Ume6. The transcriptional regulators, Nrg1 and Ume6, are of significant importance as their overexpression is shown to increase the number of sprawling cells actively released from the biofilm (Araújo et al., 2017).

In the biofilm maturation phase, ECM production is also extremely important. The gene responsible for glucan synthesis is FKS1, which is involved in resistance to fluconazole in *C. albicans* and promotes biofilm maturation in the presence of high glucose levels in *C. parapsilosis*. RLM1 and ZAP1 are the other two regulatory genes involved in matrix formation in *C. albicans*. Some ZAP1 target genes are CSH1 and IFD6 (negative regulators), and GCA1, GCA2, and ADH5 (positive regulators) that are associated with β-1,3-glucan production from the biofilm matrix. RML1 is a positive regulator, and its deletion reduces the matrix levels significantly. Other glucan regulatory genes associated with matrix formation are BGL2, PHR1, XOG1, CCR4, GAS1, GAS2, and GAS5 (Araújo et al., 2017).

The last step in biofilm formation is the detachment and dispersion of cells or parts of the biofilm, which further get displaced and colonize other parts of the host. The lack of nutrients or changes in the environment can favor this phase, which then leads to the development of infection in other organs. For this step, there are three genes involved: PES1, UME6 and NRG1. NRG1 is a negative regulator of filamentation and UME6 is necessary for the extension of hyphae (Beitelshees et al., 2018).

The main genes implicated in the genetic control of biofilm formation in *Candida* spp. are collated in Table 1.

**TABLE 1:** The main genes involved in biofilm formation in *Candida* spp.

Type	Gene	References
Adhesion	ALS1	Finkel et al., 2012; Holland et al., 2014
	ALS3	Finkel et al., 2012; Holland et al., 2014; Mendelsohn et al., 2017; Freire et al., 2018; Su et al., 2018
	ALS5	Finkel et al., 2012
	ASL9	Holland et al., 2014
	AWP	Finkel et al., 2012
	BCR1	Guan et al., 2013; Inglis and Sherlock, 2013; Holland et al., 2014; Orsi et al., 2014; Modrzewska and Kurnatowski, 2015; Marak and Dhanashree, 2018; Burgain et al., 2019; Valotteau et al., 2019
	BUD2	Valotteau et al., 2019
	CHS2	Valotteau et al., 2019
	CKA2	Valotteau et al., 2019
	CSH1	Gulati and Nobile, 2016
	CZF1	Burgain et al., 2019
	EAP1	Finkel et al., 2012
	ECE1	Holland et al., 2014; Mendelsohn et al., 2017; Freire et al., 2018; Su et al., 2018
	ECM1	Orsi et al., 2014
	ECM331	Holland et al., 2014
	EFG1	Orsi et al., 2014; Mitchell et al., 2015; Mendelsohn et al., 2017; Dominguez et al., 2018; Marak and Dhanashree, 2018; Burgain et al., 2019
	EPA1	Finkel et al., 2012; Vandeputte et al., 2012
	EPA2	Vandeputte et al., 2012
	EPA3	Vandeputte et al., 2012
	EPA4	Vandeputte et al., 2012
	EPA5	Vandeputte et al., 2012
	EPA6	Finkel et al., 2012; Freire et al., 2018
	EPA7	Finkel et al., 2012; Vandeputte et al., 2012
	GAT2	Valotteau et al., 2019
	HGC1	Mendelsohn et al., 2017
	HWP1	Finkel et al., 2012; Holland et al., 2014; Freire et al., 2018; Su et al., 2018; Valotteau et al., 2019
	HYR1	Vandeputte et al., 2012; Holland et al., 2014
	PBR1	Finkel et al., 2012
	PGA10	Finkel et al., 2012
	RBT5	Holland et al., 2014
	RSR1	Valotteau et al., 2019
	SCS7	Valotteau et al., 2019
	SNF5	Inglis and Sherlock, 2013; Modrzewska and Kurnatowski, 2015; van Wijlick et al., 2016
	TEC1	Valotteau et al., 2019
	UBI4	Valotteau et al., 2019
	UME6	Ganguly et al., 2011; Gulati and Nobile, 2016; Su et al., 2018; Burgain et al., 2019; Valotteau et al., 2019
Biofilm formation	ADH5	Gulati and Nobile, 2016
	ALG11	Mitchell et al., 2015
	BIG1	Mitchell et al., 2015
	FKS1	Gulati and Nobile, 2016; Navarro-Arias et al., 2016; Jiang et al., 2018; Leiva-Peláez et al., 2018
	GCA1	Nett et al., 2011; Gulati and Nobile, 2016
	GCA2	Nett et al., 2011; Gulati and Nobile, 2016
	IFD6	Gulati and Nobile, 2016
	KRE5	Mitchell et al., 2015; Navarro-Arias et al., 2016; Jiang et al., 2018; Leiva-Peláez et al., 2018
	MNN11	Mitchell et al., 2015
	MNN4-4	Mitchell et al., 2015
	MNN9	Mitchell et al., 2015
	PMR1	Mitchell et al., 2015; Navarro-Arias et al., 2016; Jiang et al., 2018; Leiva-Peláez et al., 2018
	RAP1	Vandeputte et al., 2012
	RIF1	Vandeputte et al., 2012
	RM11	Feng et al., 2016; Tan et al., 2017
	SIR Complex	Vandeputte et al., 2012
	SWI/SNF	Vandeputte et al., 2012
	VAN1	Mitchell et al., 2015
	VRG4	Mitchell et al., 2015
	YKU70	Vandeputte et al., 2012
	YKU80	Vandeputte et al., 2012
Morphogenesis	NTD80	van Wijlick et al., 2016; Marak and Dhanashree, 2018; Min et al., 2018; Burgain et al., 2019
	REP1	Glazier et al., 2017; Min et al., 2018
	RON1	Min et al., 2018
Oligopeptide transporter genes	OPT	Ni et al., 2009
Resistance	CDR1	Pohlers et al., 2017; Chowdhary et al., 2018
	CDR2	Pohlers et al., 2017; Chowdhary et al., 2018
	MDR1	Glazier et al., 2017
	TPO1_2	Pohlers et al., 2017
Transcriptional regulatory genes	ACE2	Guan et al., 2013; Inglis and Sherlock, 2013; Modrzewska and Kurnatowski, 2015; Burgain et al., 2019
	BGL2	Gulati and Nobile, 2016
	BRG1	Bandara et al., 2013; Freire et al., 2018; Marak and Dhanashree, 2018; Burgain et al., 2019
	CCR4	Gulati and Nobile, 2016
	CPH1	Connolly et al., 2013; Yano et al., 2019
	CPH2	Burgain et al., 2019
	CST6	Mendelsohn et al., 2017; Freire et al., 2018
	GAS1	Gulati and Nobile, 2016
	GAS2	Gulati and Nobile, 2016
	GAS5	Gulati and Nobile, 2016
	GWP1	Mendelsohn et al., 2017
	GZF3	Burgain et al., 2019
	NRG1	Ganguly et al., 2011; Gulati and Nobile, 2016; Su et al., 2018
	PES1	Ganguly et al., 2011; Gulati and Nobile, 2016
	PHR1	Gulati and Nobile, 2016
	RCA1	Mendelsohn et al., 2017
	RFG1	Su et al., 2018
	RLM1	Gulati and Nobile, 2016
	ROB1	Marak and Dhanashree, 2018; Burgain et al., 2019
	TEC1	Orsi et al., 2014; Marak and Dhanashree, 2018; Burgain et al., 2019
	XOG1	Gulati and Nobile, 2016
	ZAP1	Feng et al., 2016; Gulati and Nobile, 2016; Pierce et al., 2017; Tan et al., 2017

### Proteomics of the Biofilms

Biofilm associated proteins (55% of the biofilm dry weight) are mainly glycoproteins, glycolytic enzymes, and heat shock proteins. Around 565 different proteins have been identified, representing a total of 458 different biochemical activities (Thomas et al., 2006). Many of them are categorized as secretion signals, but most of these proteins do not have any such signal, indicating a non-canonical secretion pathway and/or protein increment occurring after cell death. In addition, host proteins including proteins related to the heme group and inflammatory and leukocyte-associated proteins, like hemoglobin, myeloperoxidase, C-reactive protein, and alarmin S100-A9 *have also been found in vivo* (Nett et al., 2015).

According to Hirota et al. (2017), the extracellular matrix of *C. albicans* is composed of extracellular DNA (eDNA), which has a crucial role in *Candida* biofilm formation and its structural integrity, and promotes the morphological transition from yeast to hyphal growth form during *C. albicans* biofilm development. The eDNA is mostly made up of random non-coding sequences and might also have an important role in antifungal resistance.

The adhesins of the fungal wall are modular proteins, and their precursors possess signal peptides to enter in the ER and GPI anchors. The mature proteins possess an N-terminal domain, which generally determines the ligand binding specificity of adhesins and is followed by a low complexity domain that in most cases contains internal tandem repeats. These internal tandem repeats have an important role in the adhesion of the fungal cells and exposure of the ligand-binding domains, which is modulated by the number of repeated copies, and consequently by the size variations of the adhesin-encoding genes identified in the clinical isolates (Willaert, 2018). According to Wagener et al. (2012), there are numerous phenotypic variations between the clinical isolates of *C. glabrata* in their ability to adhere to the abiotic surfaces for medical importance.

As high expression of heat shock proteins encourages *Candida* yeast–hyphae switch, which is an essential step in biofilm development, the expression of Hsp genes during *C. albicans* biofilm formation has been investigated previously (Becherelli et al., 2013). In contrast, heat shock protein 90 (Hsp 90) has also been shown to be involved in the dispersion process. The overexpression of the same leads to a decrease in the number of dispersed cells that are released and induction of the filamentation process. The authors recognized 226 *C. albicans* Hsp90 genetic interactors under planktonic conditions, of which 56 were identified to be implicated in transcriptional regulation. Six of these transcriptional regulators have previously been involved in biofilm development, proposing that the genetic interactors of Hsp90 found in planktonic conditions might have functional significance in biofilm formation. They further studied the relationship between Hsp90 and five of these transcription factor genetic interactors: BCR1, MIG1, TEC1, TUP1, and UPC2 (Diezmann et al., 2015).

The overexpression of Ywp1 protein in the cell wall makes the biofilm more adherent, as it negatively regulates the dispersion process. It has previously been shown that Ywp1 can collaborate biofilm detachment in early stages. On the contrary, it can contribute to biofilm maintenance during the late phases of biofilm growth. It has been proposed that Ywp1 interacts with other C. albicans adhesin proteins expressed in early, but not late phases of biofilm growth (Karkowska-Kuleta et al., 2019).

The process of cell dispersion begins early and occurs during the development phase of the biofilm. These dispersed cells have differences in their transcriptome that confer improved virulence characteristics and drug resistance, as well as a superior expression of transporters necessary for the achievement of nutrients. However, initial adhesion and maintenance are keys to biofilm biomass development. The filamentation process is important for the expression of proteins that maintain adhesion, and thus the deletion of individual proteins in *C. albicans*, such as Efg1 and Bcr1, resulted in an almost complete loss of initial adhesion and a collapse of biofilm development. However, the suppression of the expression of the ALS1 and ALS3 adhesins and Hyr1 lead to a shortage of adhesion and biofilm biomass. The hyperfilamentous strains generated by the suppression of Hog1 and Sfl1, formed a more robust biofilm. In addition, in a previous study, it was found that *C. albicans* that lacks Ywp1 protein had a weak adhesion maintenance force, but its effect on initial binding was minimal. This finding indicates that Ywp1 protein interferes with other *C. albicans* adhesive proteins (McCall et al., 2019).

The proteins expressed in *Candida* spp. biofilm formation are summarized in Table 2.

**TABLE 2:** Differential expression of the proteins involved in biofilm formation in *Candida* spp.

Type	Protein	References
Adhesion	Adhesins	Nett et al., 2015; Hirota et al., 2017
	Als1	Diezmann et al., 2015
	Als3	Diezmann et al., 2015
	Bcr1	Diezmann et al., 2015
	Efg1	Diezmann et al., 2015
	Hog1	Diezmann et al., 2015
	Hyr1	Diezmann et al., 2015
	Sfl1	Diezmann et al., 2015
	Ywp1	Becherelli et al., 2013; Diezmann et al., 2015
Biofilm associated	Alarmin S100-A9	Beitelshees et al., 2018
	C-reactive protein	Beitelshees et al., 2018
	eDNA	Thomas et al., 2006
	Hemoglobin	Beitelshees et al., 2018
	Myeloperoxidase	Beitelshees et al., 2018
Heat shock	Hsp 90	Wagener et al., 2012; Willaert, 2018

### Quorum Sensing (QS)

Quorum sensing or “quorum perception” designates a complex intercellular communication system, wherein the microorganisms coordinate to generate a uniform response for their survival and ensure the colonization of their habitats. It is the so-called “language of microorganisms” (Saxena et al., 2019).

Due to the environment, the metabolic pathways of *C. albicans* that are involved in the yeast–hypha transition, as well as its virulence, depend on a large number of molecules generated during QS (Padder et al., 2018). This process includes the production and deliverance of a signal molecule (autoinducer) that, according to cell density, will increase its concentration and favor the collective and synchronized expression of specific genes in all the species associated with biofilm formation.

According to Riekhof and Nickerson (2017), it has been demonstrated that *C. albicans* is a dimorphic fungus in the presence of farnesoic acid (FA) and farnesol (F), the two sensor molecules of related sesquiterpene quorums, and when accumulated, they do not allow the change from yeast to mycelium. Studies were conducted with three different ATCC strains of *C. albicans*, 10231, A72, and SC5314. The first strain excretes a high concentration of FA, while the remaining two secretes only F, although it is important to note that the *Candida* spp. that produce FA do produce F in undetectable amounts. In conclusion, although F and FA possess close chemical similarity, they use separate pathways to block hyphal development.

Previously, CaPHO81, a key component of the phosphate starvation response signal transduction pathway, has been shown to be implicated in inhibition of hyphal development via FA. First, it must be taken into account that the Δhot1 mutant cells used in this study, lost their sensitivity to FA but were still sensitive to F. Second, *HOT1* and *PHO81* mRNA abundance increased dramatically between 40 and 240 min after FA treatment, but these expression levels remained unchanged after F treatment as well. However, the genetic and biochemical factors that contribute to the selection for FA production versus F production are currently unexplored. Moreover, further studies will likely provide more details on the differences in virulence and immune responses between the ATCC 10231 strain used in various studies and other clinical isolates of C. albicans (Dižová and Bujdáková, 2017; Paluch et al., 2020).

Farnesol synthesized by *C. albicans* acts as a negative regulator of morphogenesis, as it inhibits the yeast–hyphae transformation. Thus, the aforesaid effect on morphological transformation via cyclic AMP (cAMP)/protein kinase A (cAMP-PKA) pathway also affects other biochemical pathways of yeasts, such as the ones for sterol biosynthesis or triggering of apoptosis via accumulation of ROS (reactive oxygen species) that damages essential cellular compartments. ROS activate intracellular caspases that indicate apoptotic response in *C. albicans* (Lindsay et al., 2012; Polke et al., 2018).

Regarding QS, we would like to highlight that Paluch et al. (2020) investigated the quorum quenching (QQ) technique and highlighted its importance because it interrupts the microbial communication and ultimately, the biofilm formation. QQ-driving molecules can decrease or even completely inhibit virulence factor production, and consequently, biofilm formation. One of the strategies is to use structural analogs of the QS receptor autoinducers. Most QQ molecules are enzymes with the ability to degrade the signaling molecules or cascades. The techniques that are being used to measure QS/QQ are mass chromatography-spectroscopy, bioluminescence, chemiluminescence, fluorescence, electrochemistry, and colorimetry. The importance of these research methods lies in their medical and biotechnological application.

## *Candida* Biofilm Models

To determine whether *Candida* spp. can form biofilms on vaginal mucosa, the use of *in vivo*, *ex vivo*, and *in vitro* models are essential. Studying the implications of *Candida* spp. in clinical practice may help in the discovery of new therapeutic targets in *Candida* spp.

### *In vitro* Models

To facilitate the detection of compounds that are active against biofilms, high-performance biofilm models are needed. Tsui et al. (2016) used a microtiter plate model to evaluate the variability between *C. albicans* biofilms formed in independent wells of the same microtiter plate. The biofilms that constituted over a 24 h period showed consistent metabolic activity.

Nweze et al. (2012) and Almshawit et al. (2014) used the Calgary Biofilm Device (CBD) for the understanding of *Candida* spp. biofilms. The CBD was also used to study the susceptibility of *Candida* spp. biofilms to metal ions and chelating agents (Harrison et al., 2007a) and identify persistent cells in such biofilms (Harrison et al., 2007b).

Samaranayake et al. (2005) used a perfusion model involving three commonly used antifungal agents, amphotericin B, fluconazole, and flucytosine, and biofilms established on microporous filters by *C. albicans*, *C. parapsilosis*, and *C. krusei*. The authors found that biofilm growth is dependent both on the antimycotic agent used and *Candida* spp. Shao et al. (2015) described a free-flow incubator that allowed biofilm organization under continuous flow conditions. The system displayed stability and continuity during the 96 h experiment. *C. albicans* and *C. glabrata* might co-exist in the dual-species biofilms under the flow regime.

Catheter-associated infections can be caused by biofilms formed on catheter surfaces. Within the biofilm, cells are protected from the host immune system. Authors studied the formation of *C. albicans* biofilms on catheters, including latex urinary catheters, and with artificial urine and antimicrobial therapies (Negri et al., 2011).

Recently, Srinivasan et al. (2012) have developed a *C. albicans* biofilm chip microarray system (CaBChip). The system is composed of more than 700 uniform and independent nano-biofilms encapsulated in a collagen matrix and is the first miniature biofilm model for *C. albicans*. In spite of multiple miniaturizations, the biofilms formed on the chip had phenotypic features that were similar to those of biofilms organized *in vitro*, containing a combination of yeast, pseudohyphae, and hyphal cells, and high concentrations of antifungal compounds. The models will allow the identification of possible anti-biofilm drugs.

The use of SkinEthic^TM^ reconstituted human epithelia models is based on the fact that the epithelial cells are placing in inert filter substrates that rise to the air–liquid interface in a humidified air incubator. Nutrient medium is added to feed the basal cells through the filter substrate. After the first 5 days, a stratified epithelium similar to human tissue is formed. The oral and vaginal epithelial tissues formed express all the major natural markers of epithelial basement membrane and epithelial differentiation, behave like human epithelium, and reflect natural wound resolution processes *in vivo*. Damage can be visualized by histopathological examination and precisely analyzed using available techniques. Confocal laser microscopy is being used (Schaller et al., 2003, 2005), although no conclusive data have yet been published.

Samaranayake et al. (2005) and other authors have verified that *Candida* spp. biofilms on mucosal surfaces exhibit characteristics similar to those of cells growing on abiotic surfaces. Murine models with vaginal yeast infections established on mucosal surfaces have demonstrated that *Candida* biofilms harbor many yeast, hyphae, and extracellular material.

Table 3 shows the main studies on *Candida* biofilm models conducted *in vitro*.

**TABLE 3:** Schema for *in vitro Candida* spp. related to biofilm models.

*In vitro* models	Components	Characteristics
Plastic/microtiter plates (Tsui et al., 2016)	Polystyrene surfaces at different temperatures (10, 20, and 37°C), flat-bottomed 96-well microtiter plates, and plastic slides	Useful for biofilm formation for different *Candida* spp. strongly associated with the type and phenotypic behavior of the isolates
Calgary Biofilm Device (CBD) (Harrison et al., 2007a; Nweze et al., 2012; Almshawit et al., 2014)	CBD was developed from polypropylene microcentrifuge tubes and pipette type boxes, as well as 96-well polystyrene pegs/plates	A useful, simple, low cost miniature device for parallel study of *Candida* biofilms and factors modulating this phenomenon.
Microporous membrane filters (Gu et al., 2015)	Microporous polycarbonate (25-mm diameter)	Quantitative evaluation of the antifungals that diffused into the disk through the biofilm
Flow system biofilm models (Shao et al., 2015)	Automated microfluidic device under laminar flow conditions	Used to study biofilm formation in real-time. The flow of liquids can influence nutrient exchange and the structural integrity of biofilms.
Catheters (Negri et al., 2011)	Silicone, polyurethane, and latex urinary catheters, with artificial urine	Used under flow conditions to study *Candida* spp. adhesion and biofilm formation
Robotic microarrayer is used to dispense yeast cells of *C. albicans* onto a solid substrate. (CaBChip) (Srinivasan et al., 2012, 2013)	CaBChip composed of ∼750 equivalent and spatially distinct biofilms with cell-based microarray platform allows for miniaturization of microbial cell culture and is fully compatible with other high-throughput screening technologies	The main advantages of the fungal biofilm chip are automation, miniaturization, savings in amount and cost of reagents and analyses time, as well as the elimination of labor intensive steps. This chip significantly speeds up the antifungal drug discovery process.
Reconstituted human epithelia (RHE) models (Schaller et al., 2003, 2005)	Epithelial cells are seeded on inert filter substrates that are raised to the air-liquid interface in a humidified air incubator	Epithelial damage can be visualized by histological analysis of the embedded and quantified based on the extracellular activity of lactate dehydrogenase (LDH) in the culture medium released by the damaged epithelial cells. Additionally, microscopy. fluorescence-activated cell sorting. ELISA can be used to measure and detect protein expression, and real-time *immune-*PCR (used to show them)

### *In vivo* Models

Experimental animal models are crucial to completely understand the candidate factors of pathogenesis and develop new therapeutic approaches, mainly in light of the increased incidence of fungal infections.

Rodents are one of the most useful animals to study candidiasis, since the disease process and host immune responses are similar to those of humans. A well-established estrogen-dependent mouse model has been developed for the study of vaginitis (Yano and Fidel, 2011). Even though, unlike humans, laboratory rodents do not naturally have *C. albicans* as a commensal, the experimentally induced infection resembles human infection. Therefore, the investigations of the animal model are translatable to the human host (Peters et al., 2014).

Wang and Fries (2011) made a murine model to investigate catheter-associated urogenital tract infections and candiduria. In this model, a guidewire is inserted through the urethra of a female mouse and a catheter segment is inserted over the guidewire into the bladder. While mucosal biofilms share many characteristics with the device-associated biofilms, whether they exhibit the same degree of resistance to antifungal compounds is unclear. Clinically, mucosal biofilms respond more frequently to antifungal therapies, including azoles (Wang and Fries, 2011).

After 5–7 days, the animal is infected intravascularly with *C. albicans*. The infection persists for approximately 28 days. During this time period, a dense biofilm of adherent yeast and hyphae forms on the luminal and extraluminal surfaces. The difficulty is that, since there is only one catheter segment, the flow will probably be less. This system can also be used in other mammalian animals, including wild or immunocompromised animals.

Since there is only one catheter segment in place in the model, the flow conditions are probably less than what would be seen for a catheter installed in a patient, which functions to drain the bladder. The model includes the mammalian immune system. In addition, the model may use wild type or immunocompromised animals. To the best of our knowledge, this model has not been used to investigate the activity of anti-biofilm therapies yet.

Naglik et al. (2008) administered 0.1 mg 17-β-oestradiol in 0.1 ml sesame seed oil subcutaneously for 3 days to female mice prior to intravaginal inoculation with 20 μl *C. albicans* 3153A (2.5 × 10^6^ cells/ml), *C. albicans* DAY185 (2.5 × 10^8^ cells/ml), or biofilm mutant (2.5 × 10^8^ cells/ml) suspension. After a period of time, the mice were euthanized. The vagina was removed from each mouse and examined through confocal or scanning electron microscopy.

Harriott et al. (2010) performed an *ex vivo* investigation that involved female mice treated with estrogen. After euthanizing, the vagina was removed from each mouse. *C. albicans* isolates (1 × 10^6^ blastoconidia) in 0.1 ml PBS was incubated at 37°C in an atmosphere of CO_2_ and instilled on the vaginal surface. The fungal load was determined and microscopy examination was performed.

Another recently used model for the *in vivo* study of *Candida* spp. biofilms is the nematode *Caenorhabditis elegans*. The model is relevant for aspects of human infections, including resistance to antifungal agents (Madende et al., 2020).

The most relevant studies on *Candida in vivo*/*ex vivo* biofilms are summarized in Table 4

**TABLE 4:** Schema for *in vivo/ex vivo Candida*-associated urogenital biofilm models.

*In vivo* models	Device	Animal species	Characteristics	References
Catheter-associated models	Candiduria model: a subcutaneous foreign body system featuring a catheter through the urethra of a female mouse	Rat, mouse, and rabbit	Rat and mouse models have advantages over rabbit models that include relatively low cost, ease of use, and ability to mimic the clinical conditions of rabbit models	Wang and Fries, 2011; Yano and Fidel, 2011; Peters et al., 2014
Models in which the vagina of each animal is excised and cut longitudinally to expose the mucosal surface.	17-β-Estradiol subcutaneous + intravaginally administered *C. albicans*	Female mice	Tissue is used to determine fungal load through confocalor scanning electron microscopy	Naglik et al., 2008
Models using biotic surfaces, such as vaginal mucosa	*Ex vivo* models	Mouse (treated with estradiol prior to infection)	Low cost and mimics clinical conditions	Harriott et al., 2010
Animal is infected with fungi by consuming the yeast cells as a food source	*In vivo* models	Nematode *Caenorhabditis elegans*	Identification of antifungal chemical compounds	Madende et al., 2020

### Biofilm Inhibition

It should be noted that despite the existence of numerous investigations, no specific drugs are available for treating biofilm-produced VVC/RVVC yet.

Posaconazole shows *in vitro* and *in vivo* synergy with caspofungin against *C. albicans*, including echinocandin-resistant isolates. Furthermore, an *in vitro* study of *C. albicans*, *C. glabrata*, and *C. parapsilosis* showed that azole-resistant *Candida* spp. are not resistant to the combination of both compounds (Cui et al., 2015). Similar results were obtained by Ning et al. (2015) with epigallocatechin gallate bound to azoles.

In a study of the various biological properties of the thiazolidinone scaffold, 100 compounds were synthesized and characterized by a 1,3-thiazolidin-4-one nucleus derivatized at the C2 with a hydrazine bridge, linked to (cyclo) aliphatic or hetero (aryl) moieties. The *N*-benzylated derivatives of these compounds were found to be highly effective against *Candida* spp. (Carradori et al., 2017).

Mannich base-type eugenol derivatives have also been synthesized and shown to be particularly effective against *C. glabrata* (Abrão et al., 2015).

Piperazine derivatives 1c–34e have been synthesized from phenol, 2,4-dichlorophenol and 4-hydroxybiphenyl. Their inhibitory activity during hyphae formation was verified and it correlated with the inhibition of biofilm formation by *C. albicans*, thus possibly indicating the vital role of hyphae development in the *C. albicans* biofilm formation process (Zhao et al., 2018).

A new series of glycosides modified in their saccharide units has been analyzed against *Candida* sp. The newly synthesized glycoside is called eugenol glucosiso 5 and can be considered a new structural pattern in anti-*Candida* drugs, fundamentally against *C. glabrata* (de Souza et al., 2016).

When a cationic peptide antibiotic was used in combination with antifungal agents, such as polymyxin B with azoles, a synergist effect was seen (Pankey et al., 2014; Raman et al., 2015). Aminoglycosides, like tobramycin, have been shown to possess antifungal potential (Fosso et al., 2018). Silva et al. (2014) showed that cerium nitrate has fungicidal activity against planktonic *Candida* cells. Similarly, the ability of cerium nitrate to pare and disarticulate biofilms has been demonstrated, which represents an important advance in clinical practice. Thamban Chandrika et al. (2018) identified fluconzaol analogs and found that the antifungal activity of alkyl-amino fluconazole derivatives depends on the alkyl chain length. Recently, compounds 6–9 were identified as promising antifungal agents, with low cytotoxicity and hemolytic activity. These compounds have higher activity levels and lower toxicity than fluconazole.

Antifungal lock therapy is used to inhibit biofilm formation. Micafungin (5 and 15 mg/L), especially combined with ethanol; capsofungin (5 and 25 mg/L); and posaconazole (10 mg/L) have been used in this form of therapy (Walraven and Lee, 2013). Liposomal amphotericin B (5 mg/mL) or anidulafungin (3.33 mg/mL) has also been successfully used (Basas et al., 2019).

Nikkomycin Z has been shown to act synergistically in association with caspofungin or micafungin against biofilms (Kovács et al., 2019).

Given the resistance of fungi to fluconazole, new compounds derived from this antifungal compound have been investigated. A total of 27 new fluconazole derivatives have been tested for their antifungal activity against a panel of 13 clinically relevant fungal strains (Shrestha et al., 2017).

Classically, the combination of antifungals and antibiotics, such as rifampicin, have been shown to have a general role in regulating signal transduction or modulating gene expression by other mechanisms in *C. albicans* (Vogel et al., 2008). Similarly, synergistic interactions between fluoroquinolones and antifungal agents (amphotericin B or caspofungin) have been demonstrated, potentially improving the outcome in immunosuppressed patients with concurrent bacterial and fungal infections (Stergiopoulou et al., 2009). Recently, chloramphenicol has been shown to have activity, mainly against *C. albicans* and C. *glabrata* (Joseph et al., 2015). The combination of antifungals and QS molecules is also of interest. Farnesol, a QS molecule, inhibits the formation of hyphae and biofilms, and tyrosol and farnesol are excellent antifungal candidates (Monteiro et al., 2017; Mehmood et al., 2019).

Using nanotechnology, amphotericin B-loaded silver nanoparticles have been developed and shown promise in improving the antifungal capacity of amphotericin B against *Candida* spp. (Leonhard et al., 2018).

Some success has also been obtained with 0.43–1.736 mM acetylsalicylic acid use. It is especially effective due to its synergistic effects with amphotericin B (Zhou et al., 2012). Ibuprofen (Sharma et al., 2015) and ambroxol (Li et al., 2017) have also been successfully used as antifungal agents.

Synthetic compounds, such as the peptides, KSL-W and SM21, which inhibit the transition from yeast to hypha, also inhibit biofilm formation (Theberge et al., 2013; Cavalheiro and Teixeira, 2018).

Photodynamic therapies using light of a certain wavelength and a photosensitizing dye are also being investigated. These therapies produce reactive oxygen species (ROS), and after treatment, substances such as hydrogen peroxide can be used. The absence of dye toxicity and low cost of the technique suggest that this alternative approach has great potential (Bujdáková, 2016).

Polymers incorporated in medical devices can also be modified to prevent fungal contamination. Lumbrical organ water-insoluble polyethylamine derivatives are capable of inhibiting *C. albicans* growth, by altering the membrane integrity. Furthermore, the natural polymer, chitosan, is also effective against *C. albicans* biofilms (Hoque et al., 2015).

Table 5 shows the main advances in new compounds involving new therapeutic options directed toward fungal biofilms.

**TABLE 5:** New anti-*Candida* spp. biofilm compounds.

Compound	Action	References
Posaconazole plus caspofungin (*in vitro* and in animals) or Concomitant use of epigallocatechin gallate and miconazole, fluconazole, or amphotericin B	*C. albicans*, *C. glabrata*, and *C. parapsilosis* Highly effective against *C. albicans, C. glabrata* and *C. parapsilosis in vitro* and in animal experiments	Cui et al., 2015 Ning et al., 2015
1,3-thiazolidin-4-one nucleus and its N-benzylated derivatives at the C2 with a hydrazine bridge linked to (cyclo)aliphatic or hetero(aryl)	Strong activity against *Candida* spp. Lack of cytotoxic effects	Carradori et al., 2017
Mannich base-type eugenol derivatives: : 4-allyl-2-methoxy-6- (morpholin-4-ylmethyl) phenyl benzoate (7) and 4- {5-allyl-2 - [(4-chlorobenzoyl) oxy] -3-methoxybenzyl}. Morpholin-4-io (8) chloride was found	Highly effective against *C. albicans, C. glabrata*, and *C. krusei*	Abrão et al., 2015 Yano et al., 2012
1-(4-ethoxyphenyl)-4-(1-biphenylol-2-hydroxypropyl)-piperazine	Acts primarily on *C. albicans* Low cytotoxic effects	Zhao et al., 2018
Glucosides with modified saccharides	Fungistatic activity against *C. glabrata*	de Souza et al., 2016
Amphiphilic, helical β-peptide structural mimetics of natural antimicrobial α-peptides	Specific planktonic antifungal and anti-biofilm activity against *C. albicans, C. glabrata, C. parapsilosis*, and *C. tropicalis*	Pankey et al., 2014 Raman et al., 2015
Aminoglocosides derived from tobramycin	The triazole is most effective against *Candida* spp.	Fosso et al., 2018
Cerium nitrate, a member of the lanthanide family	Active against planktonic and sessile *Candida* spp. cells It is able to prevent biofilm formation by *C. albicans* and *C. parapsilosis* both *in vitro* and *in vivo* Application in medical devices	Silva-Dias et al., 2015
Fluconazole analogs with alkyl-, aryl-, cycloalkyl-, and dialkyl-amino substituents	These compounds are active against some of the *C. albicans* and non-*albicans Candida* strains and are highly effective against clinical strains of *C. glabrata* and *C. parapsilosis*	Thamban Chandrika et al., 2018
Micafungin + ethanol Capsofungin/posaconazole, or amphotericin B, or anidulafungin, or Caspofungin/micafungin in combination with nikkomycin Z	Antifungal lock therapy is used to inhibit the formation of the biofilm	Walraven and Lee, 2013 Basas et al., 2019 Kovács et al., 2019
27 new FLC derivatives	Broad-spectrum antifungal activity. All compounds inhibit the sterol 14α-demethylase enzyme involved in ergosterol biosynthesis	Shrestha et al., 2017
Fluoroquinolones and antifungal agents (from amphotericin B or caspofungin) or rifampicin Chloramphenicol	Very useful in immunosuppressed patients	Stergiopoulou et al., 2009 Vogel et al., 2008
	Not valid for use against *C. albicans* or *C. glabrata* Antifungal activity comparable to caspofungin and ketoconazole	Joseph et al., 2015
Tyrosol and farnesol	Strong biofilm inhibition Inhibit the formation of hyphae	Monteiro et al., 2017 Mehmood et al., 2019
Amphotericin B plus silver hybrid nanoparticles	Powerful antifungal activity although the toxicity of the nanoparticles depends on the size, concentration, and pH of the medium and the exposure time to pathogens	Leonhard et al., 2018
Amphotericin B plus acetylsalicylic/ibuprofen/ambroxol	They are inexpensive, but they increase the risk of bleeding and hyperkalaemia	Zhou et al., 2012 Sharma et al., 2015 Li et al., 2017
KSL-W and SM21 peptides	Inhibit biofilm formation by *Candida* spp.	Theberge et al., 2013 Cavalheiro and Teixeira, 2018

### Immune Response Intervention

Peptide-derived antibody therapies against *Candida* spp. are currently being developed. These therapies increase the oxidative stress of the yeast and membrane permeability to significantly decrease the expression levels of biofilm generation-related genes (Paulone et al., 2017).

New targets are also being investigated to develop vaccines for RVVC. An effective RVVC vaccine is a possible option to treat this chronic condition. An ideal vaccine should be able to induce an efficient immune response that promotes fungus elimination and virulence factor neutralization, without causing harmful changes in the vaginal microenvironment (Cassone, 2015). Vaccines that target the adhesin, Als3, and the enzyme, Sap2, have been developed. Although no vaccine has been approved for use in humans currently, important advances in this area are expected in the near future (Sui et al., 2017; Tso et al., 2018). In a recent study, 70 women of fertile age were recruited and divided into two groups. One group was treated by intravaginal autolymphocyte therapy, in conjunction with traditional treatment, and a control group only received conventional treatment. The group of patients treated by autolymphocyte therapy recovered fully in half the time than that required by the control group (Alyautdina and Esina, 2019).

### Probiotics

The term probiotics refers to microorganisms with beneficial actions on the human body. To be classified as a probiotic, a microorganism must be correctly identified at the genus, species, and strain levels using phenotypic and genotypic methods, since the demonstrated beneficial effects of a specific strain cannot be extrapolated and attributable to another strain of the same species. The strain is also required to be deposited in an internationally recognized collection. Probiotics should lack virulence factors and/or the ability to produce metabolites that are undesirable for the host. The main objective of probiotic therapy is not to re-establish the vaginal canal bacterial microbiome; and there is no consensus for its use in vaginal infection treatment or their sequelae (Hewadmal and Jangra, 2019). However, Rodríguez-Cerdeira et al. (2019) have extensively reported the importance of probiotics in VVC/RVVC treatment. Ongoing research on this topic and recently performed studies are discussed below.

Tsimaris et al. (2019) studied a group of 70 women treated with *Lactobacillus* during the pre-diagnosis period. Administration of intravaginal *B. coagulans*, alone and in combination with standard antibiotic therapy, provided significant benefits in the treatment of vaginal symptoms in VVC patients in this clinical trial.

Previous studies support the notion that vaginal microbiota restoration and/or local mucosal immune response modulation can be achieved via supplementation with probiotics, which can be administered orally as a probiotic food supplement, intra-vaginally as vaginal suppositories, or applied topically as a gel (Köhler et al., 2012; Gille et al., 2016).

Gabrielli et al. (2018) have verified the probiotic capacity of *Saccharomyces cerevisiae* CNCM I-3856 to modulate the expression of *C. albicans* pathogenicity in mice with vaginal candidiasis. Daily intravaginal administration of *S. cerevisiae* CNCM I-3856 was shown to eliminate various components of the fungus necessary for its virulence in the vagina and modulate the expression of aspartyl proteinase and genes associated with hyphal growth, *Hwpl* and *Ecel*, in the epithelium. The decreased inflammatory response observed in this study was likely due to the decrease in IL-8 production and aspartyl proteinase expression inhibition. Despite the assumption that *S. cerevisiae* administration would produce undesirable effects, it did not alter the architecture of vaginal epithelial cells or organs, either *in vitro* or in the human vaginal epithelium *in vivo* (Handalishy et al., 2014; van de Wijgert and Verwijs, 2020).

In general, the studies reported contradictory results and probiotics were generally not effective in preventing or curing VVC. None of the studies showed safety issues with the tested probiotics. There was no evidence of vaginal colonization by the probiotic strains and thus, vaginal detection is restricted to the dosing period. It should be noted that the different probiotic products used in these studies differ in their components, such as the active ingredients and excipients, and the application methods and dosages differ between the different studies. There is also no consensus on which specific strains of *Lactobacillus* may be most beneficial for dysbiosis. However, the efficacy of the different probiotics analyzed in the studies was highly similar.

## Discussion

The importance of the present review lies in the fact that it raises awareness regarding the impact of biofilms on practical clinical management and treatment of VVC/RVVC and highlights the need for additional research toward the development of novel therapeutics targeting pathogenic vulvovaginal biofilms.

As discussed in the present review, numerous transcriptional events related to morphogenesis, key molecule expression, and virulence factor manifestation occur during the invasion and infection processes. Experimental evidence indicates that *C. albicans* can differentially regulate its genes during adaptation to and subsequent colonization of a biological niche, and it exhibits a specific profile of virulence factors depending on the type of mucosa in which the infection occurs. In this review, we focussed on the mucosa of the lower genital tract (TGI) with a special emphasis on the vulvovaginal mucosa (El-Houssaini et al., 2019).

*Candida* spp. that form biofilms exhibit high resistance to antifungal drugs and are known to successfully evade host defense mechanisms. This resistance is responsible for the perpetuation and reappearance of this type of infection, causing collateral damage to the surrounding tissues.

Present interventions for preventing biofilm formation are based on attempts to modulate surface chemistries to ensure prevention of attachment (Li et al., 2018) or blocking molecules involved in signal transduction, such as c-di-GMP, from regulating attachment and matrix production (Hall and Lee, 2018).

Other substances used are proteases immobilized on a polypropylene surface, which decreases the adhesion of *C. albicans* required for biofilm formation, although the toxicity of these enzymes remains an issue (Andreani et al., 2017).

Disruption of the maturation of *Candida* biofilms with the alkaloid berberine has been successfully demonstrated in both planktonic and biofilm conditions, as described by Xie et al. (2020). The authors observed significant alterations in the architecture of biofilms formed by *C. albicans* (ATCC 10231 and ATCC 90028), *C. krusei* (ATCC 6258), *C. glabrata* (ATCC 90030), and *C. dubliniensis* (MYA 646).

The role of epithelial cell-mediated immunity of the vulvovaginal mucosa is crucial in the context of protection against vaginal *Candida* infections. The FGT is equipped with various mechanisms responsible for innate immunity that are important in maintaining immunological surveillance against microorganisms, upholding the basic tenets of commensalism, and ensuring protection against invasion (Cassone, 2018). These include neutrophil polymorphonuclear cells (PMNs), macrophages, dendritic cells, natural killer (NK) cells, T lymphocytes, and innate lymphocytes, which actively contribute to the localized antifungal response (Richardson et al., 2018; Yano et al., 2018). Most of these cells reside in the mucous membranes of the TGI, although additional molecules are recruited in response to infection. In addition to the transforming growth factor (TGF) involved in immunoregulation, epithelial cells express innate immunity receptors, such as pattern recognition receptors (PRRs) and a broad spectrum of antimicrobial peptides including alarmins, chemokines, and cytokines [such as IL-1, IL-6, IL-8, and tumor necrosis factor (TNF)]. All these factors initiate the first phase of the response and contribute to the recruitment of other cell populations (Doerflinger et al., 2014; Pericolini et al., 2015).

Epithelial cells can discern between the saprophytic and hyphal forms of *C. albicans*, leading to activation of the inflammatory response if necessary. Yeast to hypha transition and virulence factor production causes epithelial disruption and results in PMN recruitment, which, in turn, exacerbates the inflammation (Yano et al., 2018). Previous studies have identified the alarmins, S100A8 and S100A9, as key chemotactic mediators of the acute PMN response toward Candida infection (Yano et al., 2012).

According to Jaeger et al. (2013), epithelial cells express PRRs, which are capable of detecting the presence of microorganisms and sending activation signals to induce immune mediator secretion. There are 3 PRR families involved in the recognition of *Candida* pathogen-associated molecular patterns (PAMPs). The different PAMPs of *Candida* spp. recognized by these PRRs are well-characterized. Toll-like receptors (TLR)2, TLR4, TLR7, and TLR9 recognize phospholipomannans, *O*-mannoside-rich structures, *Candida* RNA, and *Candida* DNA, respectively. NOD-like receptors (NLRs) are found in the cytosol of cells. NLRP3 constitutes a part of the inflammasome, which is a cytoplasmic multiprotein complex with enzymatic activity. Other receptors, such as mannose receptor, dectin-2, DC-SIGN, and mincle recognize other glucidic structures, such as mannose and fucose, located in the *Candida* cell wall (Stoddart et al., 2011; Yano et al., 2018).

As described by Yu and Gaffen (2008), Th17 cells within the CD4^+^ T lymphocyte population secrete IL-17A, IL-17F, IL-22, and IL-263, but differ in their production of cytokines such as IL-1, IL-6, and TGF3. IL-23 is an absolute requirement for the expansion, maintenance, and effector functions of this cell population. NKT cells, T lymphocytes, and group 3 innate lymphoid cells (ILC3s) produce abundant IL-17 and play important roles in defending the mucosa (Conti et al., 2009).

Identification of the key role of morphogenic changes in fungus and production of Sap as a virulence factor that activates the local inflammatory response have been associated with the discovery of mutations and genetic polymorphisms in the PRRs (Kalia et al., 2019) and their activation pathways. These observations collectively highlight the mechanism of VVC pathogenesis.

Biofilm formation in RVVC is usually related to the host immune system, when evasion of the defense system occurs at both humoural and acquired levels. Complement activation is known to be decreased in biofilms compared to that in plankton cells, and altered phagocytosis occurs due to the surrounding extracellular matrix of polymeric substances (Duggan et al., 2015).

It is important to highlight that persister cells that remain latent inside the biofilm evade elimination by macrophages (Mina and Marques, 2016; Wuyts et al., 2018). Therefore, understanding the contributions of these biofilms in fungal burden and in shaping subsequent strategies that influence and modulate the local immunopathogenic response is imperative for the development of efficient therapeutic modalities.

Emerging therapeutic approaches have centered on harnessing the complex interaction between commensal and pathogenic organisms in the vaginal environment. This is implemented mainly by diets rich in probiotics or vaginally administered probiotics, and this has been shown to restore the vaginal microbiota and decrease the incidence of VVC/RVVC. The work of Miyazima et al. (2017) provides support to these claims.

Combinations of prebiotics and probiotics, also called symbiotics, not only have therapeutic applications in infections in the intestinal tract but also for the urogenital area (Olveira and González-Molero, 2016).

Special care is taken with infections associated with mucosal devices (Nett, 2016). In these cases, inhibition of yeast adhesion to surfaces or devices (coating surfaces) constitute an important therapeutic aspect for patients with a catheter or similar device inserted in the urogenial tract (Palmieri et al., 2018).

Novel associations, emerging drugs, and new activities are being studied in response to yeasts in *Candida* spp. biofilms, as described in the previous section (Fosso et al., 2018; Thamban Chandrika et al., 2018; Černáková et al., 2019).

The resolution of these challenges would enable important advances in understanding and therapeutic management of this mycosis. Here, we summarize the potential therapeutic targets in three parts: (a) those aimed at inhibiting or eliminating biofilms, (b) those intervening in the immune response, and (c) those mediated by probiotics.

## Conclusion

Mycological diagnosis requires a comprehensive knowledge of the complex mechanisms underlying the interaction between a fungus and its host, and the identification of fungi is not solely limited to complete and fine technical handling in the laboratory. Fungi are eukaryotic entities with a markedly more intricate physiology and pathophysiology than viruses or bacteria, and they are an increasingly frequent cause of morbidity and mortality.

Rapid and accurate identification of pathogenic yeasts is an objective in proper patient management, especially for fungal infection control. Incorporation of modern methodologies based on molecular, genomic, proteomic, and genetic engineering techniques has facilitated the identification of different strains of *Candida* spp. These methodologies have contributed toward preventing the irrational use of antifungals and selecting appropriate empirical therapy.

*Candida* spp. biofilms have considerable clinical repercussions in VVC/RVVC owing to the increasing frequency in resistance to antifungals presented by these patients. The capacity of *Candida* spp. strains to acquire resistance confers various advantages, such as the colonization of host tissues, expression of virulent characteristics, metabolic cooperation, efficient capture of nutrients, cell–cell communication, and exchange of genetic material, which provide a considerable ecological advantage, as it protects against antifungals.

Thus, the genes involved in biofilm formation and development as well as the quorum-sensing systems are considered new targets in the development of specific inhibitors as an alternative to currently available treatments.

The ability of yeasts of the genus *Candida* to generate a biofilm is multifactorial and generally considered to be dependent on the site of infection, species and strain involved, and microenvironment in which it develops. Thus, as presented in this manuscript, the virulence attribute is not exclusive to a particular *Candida* species and new strains with greater formative capacity may emerge any time.

Although the *in vitro* systems studied in the present study had certain limitations, they allowed simultaneous processing of a large number of samples, which is ideal for screening. The systems also enabled easy testing of various physicochemical and biochemical parameters as well as studying the activity of antimicrobial agents. From a genomic point of view, the systems allowed us to analyze the genes involved in biofilm regulation and formation in the strains capable of forming biofilms (*in situ*).

*In vivo* studies in murine VVC/RVVC models are commonly used to study *C. albicans* infection. Using these models, we will first investigate the virulence factors associated with *Candida* spp., including adhesins, aspartyl proteases, and hydrophobic properties. Second, we will analyze the factors favoring such infections, such as alterations in the bacterial flora. Third, we will examine the immune response to this infection in mice with genetic alterations. Finally, we can evaluate antifungal molecules through pharmacokinetic studies; prophylactic, therapeutic, and new molecule models; synergism studies; and *in vitro, in vivo*, and *ex vivo* correlation analyses.

The future of *Candida* spp. infection biology should simultaneously integrate the study of pathogenic factor analysis and host immunological characteristics to generate a comprehensive and detailed assessment of host-pathogen interactions. Therefore, the new VVC/RVVC approach aims to not only control fungal load, but also develop strategies targeted for modulating the local immunopathogenic response.

Finally, we must be particularly careful with immunocompromised patients with VVC/RVVC, as the release of new fungal cells from the biofilm may spread the infection throughout the body, which can lead to systemic candidiasis.

## Author Contributions

CR-C conceived, designed, and wrote the review. EM-H, MC-G, and AL-B provided the ideas and contributed to writing the manuscript. GF, MF, ME-S, and JG-C addressed the questions related to bibliography and summaries. All the authors have read and approved the final version of the manuscript.

## Conflict of Interest

The authors declare that the research was conducted in the absence of any commercial or financial relationships that could be construed as a potential conflict of interest.
